# Phosphatase of regenerating liver-3 (PRL-3) is associated with metastasis and poor prognosis in gastric carcinoma

**DOI:** 10.1186/1479-5876-11-309

**Published:** 2013-12-13

**Authors:** Xiaofang Xing, Shenyi Lian, Ying Hu, Ziyu Li, Lianhai Zhang, Xianzi Wen, Hong Du, Yongning Jia, Zhixue Zheng, Lin Meng, Chengchao Shou, Jiafu Ji

**Affiliations:** 1 Department of gastrointestinal translational research, Peking University Cancer Hospital & Institute, #52 Fu-Cheng Road, Hai-Dian District, Beijing 100142, China; 2 Department of Biochemistry and Molecular Biology, Peking University Cancer Hospital & Institute, #52 Fu-Cheng Road, Hai-Dian District, Beijing 100142, China; 3 Tissue bank, Peking University Cancer Hospital & Institute, Beijing, China; 4 Department of gastrointestinal surgery, Peking University Cancer Hospital & Institute, Beijing, China

**Keywords:** PRL-3, Gastric cancer, Prognosis, Metastasis

## Abstract

**Background:**

PRL-3 is a member of phosphatases of regenerating liver family, characterized by phosphatase active domain and C-terminal prenylation motif. Overexpression of PRL-3 has been implicated in multiple cancers. Here we examined the clinical significance of PRL-3 in gastric cancer together with its metastatic biological functions utilizing different structural mutants.

**Methods:**

PRL-3 expression was analyzed immunohistochemically in 196 gastric cancer patients and 21 cases of liver metastasis. A series of wild type PRL-3 or its mutant plasmids were expressed in BGC823 cells to investigate the relationship between its catalytic activity, cellular localization and metastatic potential *in vitro*.

**Results:**

Positive staining of PRL-3 was observed in 19.4% (38/196) gastric cancer tissues compared with 76.2% (16/21) in liver metastasis. Statistical analysis revealed that PRL-3 expression correlated with lymph node metastasis and vascular invasion (*P* < 0.05). Patients with high PRL-3 expression showed poorer 5-year overall survival (*P* = 0.011). Wild type PRL-3 expressing cells resulted in enhanced migration and invasion ability, which were greatly crippled in form of PRL-3(C104S) or PRL-3(ΔCAAX) mutants accompanied with its alteration in subcellular localization.

**Conclusions:**

Metastasis associated protein PRL-3 may serve as a potential prognostic biomarker in human gastric cancer. Both the phosphatase catalytic activity and cellular localization are critical for its function.

## Introduction

Despite a decrease in incidence in recent decades, gastric cancer is still the second leading cause of cancer-related death worldwide, especially for those in advanced stages with metastatic lesions that still has a rather poor outcome [1]. As clinicians move towards personalized cancer medicine, there is an urgent need to understand and identify key factors involved in the biology of metastasis, not only to predict gastric cancer outcome, but also to select a subset of population for appropriate targeted therapy before disease progression.

PRL-3 (phosphatase of regenerating liver-3, also known as PTP4A3) belongs to the the family of protein tyrosine phosphatases (PTPs). PTPs are important for regulating phosphorylation of many crucial signalling molecules and take effect on cell cycle, proliferation, differentiation and transformation [2]. Using serial analysis of gene expression (SAGE), PRL-3 was first identified as the only gene that is consistently overexpressed in all 18 liver metastases derived from colorectal cancer, but at low levels in primary tumors and normal epithelium [3]. Since then, PRL-3 overexpression has been reported to be related with the poor prognosis of multiple cancers, including colorectal cancer [4-6], breast cancer [7], lung cancer [8], ovarian cancer [9], and hepatocellular cancer [10]. Mostly, it has been found to be associated with metastasis and has been proposed as a potential biomarker for assessing tumor aggressiveness. In gastric cancer, Miskad et al. observed high expression in primary tumors and higher expression in lymph node metastasis (68.1% and 92.6%, respectively) [11]. Similar results were obtained by Li et al. [12]. However, these research were conducted utilizing polyclonal antibodies, which might have cross-reaction with other PRL family members (PRL-1 and PRL-2) considering their high homology [2]. Afterwards, Wang et al. found that overexpression of PRL-3 was present in 47.7% of gastric carcinomas with the lymph node metastasis using monoclonal antibody [13] and reported its prognostic significance [13]. Although correlation between PRL-3 overexpression and lymph node metastasis or peritoneal metastasis has been reported at some aspects in gastric cancer [12,14], the identical expression in the primary tumors without metastasis, primary tumors with metastasis, and matched samples of primary lesion and liver metastasis has not been completely understood. Also, the prognostic value of PRL-3 expression has not been reached a consensus on its clinical significance.

PRL-3 is composed of 173 amino acids and is a monomer with a complex structure [15]. Enzyme active site is located at position 103–110, where Cys104 is the enzymatic nucleophile [16]. Our previous studies have found that PRL-3 interacted with integrin α1, downregulated tyrosine phosphorylation of integrin β1, enhanced the phosphorylation of ERK1/2 and further increased the gelatinolytic activity of gelatinase MMP-2, thus finally promoted metastasis in colon cancer cells [4,17]. Some other studies also reported its prometastatic function through reconstruction of the cell cytoskeleton [18], epithelial-mesenchymal transition [19,20] and angiogenesis process [21,22]. As PRL-3 is a phosphatase, it is important to investigate whether its catalytic activity itself is directly involved in the cancer metastasis.

Moreover, PRL-3 contains C-terminal CAAX sequence for prenylation, which is a common post-translational modification for proteins that are targeted to membranes and enables participation in their signalling pathways [23]. Zeng et al. reported that PRL-3 was mainly located at plasma membrane and the early endosomes with a small fraction of unprenylated proteins in the nucleus [24]. Given that CAAX motif is not only responsible for prenylation which enables correct cellular localization, but also plays an additional role in the regulation of PRL-3 by inhibiting its catalytic activity. Here we explored the role of prenylation of the CAAX motif in PRL-3′s cellular localization and in the process of gastric cancer cell metastasis.

In the present study, we first detected PRL-3 expression in primary gastric carcinoma with or without metastasis and in 21 cases of matched liver metastases using immunohistochemistry. The aim was to evaluate the association between PRL-3 overexpression and clinical pathological factors and analyze its impact on survival. Then, prometastatic effects of wild type PRL-3 and its catalytic inactive and CAAX motif deleted mutants were observed *in vitro* in order to clarify the importance of its catalytic activity and subcellular localization for its functional role in the regulation of metastasis.

## Materials and methods

### Patients and tissue specimens

A total of 196 gastric cancer patients who underwent surgical resection from February 1998 to January 2007 at Peking University Cancer Hospital were analyzed. The records of patients were reviewed in the context of clinicopathological and follow-up information. The stage of gastric cancer was classified according to the American Joint Committee on Cancer stage (AJCC 7th edition). The OS (Overall Survival) was calculated starting from the date of the initial surgery to the time of death, counting death from any cause as the end point or the last date of follow-up as the end point, if no event was documented. All patients were followed up until November 2011. None of the patients received preoperative chemotherapy or radiation therapy. After gastrectomy, resected specimens were processed routinely for macroscopic pathological assessment. Informed consent was obtained from each patient.

### Immunohistochemistry analysis

The validation of the PRL-3 antibody 3B6 used for immunohistochemistry has been described previously [25]. Four-μm sections from formalin-fixed, paraffin-embedded tissues were mounted on poly-L-lysine-coated slides and then deparaffinized in xylene and rehydrated through graded alcohol to distilled water. Endogenous peroxidase activity was then blocked by incubation in 3% hydrogen peroxide–methanol for 10 min. After washing with phosphate–buffered saline, the slides were blocked with 5% skim milk for 60 min and then incubated with PRL-3 monoclonal antibody 3B6 [25] (5 μg ⁄ ml) overnight at 4°C. EnVision + TM (Dako, Carpinteria, CA, USA) was used as the secondary antibody. Antibody binding was visualized by a standard streptavidin immunoperoxidase reaction, followed by chromogen detection with diaminobenzidine for 10 min and haematoxylin counterstaining. Immunoreactivity in the cytoplasma and cytoplasmic membrane was evaluated.

### Semiquantitative immunohistochemical scoring

Evaluation of PRL-3 immunoreactivity was carried out independently by three experienced pathologists without any knowledge of the clinical data. All tissue samples were assessed in a consecutive analysis to ensure maximal internal consistency. The analysis was assessed according to both the percentage of positive cells and the intensity of cytoplasmic reactivity. Each histological section was examined at 40 magnification to identify areas of maximum tumour positivity. At 200 or 400 magnification, cells were analyzed from five areas of maximum tumour positivity in each case and the average percentage of positive cells was recorded. As described in our previous study [6], these averaged values were stratified into five scoring groups:-, not detected; ±, <10% positive cells; +, 10–20% weakly to moderately positive cells; ++, 10–20% intensely positive cells or 20–50% weakly positive cells; and +++, 20–50% positive cells with moderate to marked reactivity or >50% positive cells. There was a high level of consistency among the three pathologists, and in the few discrepant cases (<5%) a consensus was reached after joint review. On statistical analysis, and ± were considered negative, + and above were considered positive.

### Reagents and cell culture

Monoclonal antibody 3B6 against PRL-3 was generated as previously described [25]. Gastric cancer cell line BGC823 (ATCC, Manassas, VA) were maintained in RPMI-1640 medium (Invitrogen) supplemented with 10% fetal calf serum.

### Plasmids and transfection

Myc-tagged wild type PRL-3 cDNA [GenBank: NM_032611] was inserted into pcDNA3.1 at BamH I/Xba I sites to generate a mammalian expression plasmid pcDNA3.1-PRL-3 as previously described [4]. Then, the catalytically inactive mutant of PRL-3(C104S) was created by standard PCR based site-directed mutagenesis using the Easy Mutagenesis System (Transgene Biotech). (forward primer: CAG GCC CGC CAC AGA GTG CAC AGC; reverse primer: GCT GTG CAC TCT GTG GCG GGC CTG with the substituted nucleotides encoding serine at the site of 104 instead of cysteine. PcDNA3.1-PRL-3(ΔCAAX) was constructed by insertion of PRL-3 sequence with C-terminal CAAX motif truncated into pcDNA3.1 plasmid (forward primer: CGC GGA TCC ATG GCC CGC ATG AAC CGG, reverse primer: CCG GAATTC TCA CCG GGT CTT GTG CGT GTG TGG GTC). They were transfected into BGC823 cells with Lipofectamine 2000 (Invitrogen) to generate wild type PRL-3, PRL-3(C104S), and PRL-3(ΔCAAX) stably expressing and control cell pools, respectively. After 4 weeks of selection with 600 μg/mL of Geneticin (Invitrogen), PRL-3 expression was verified by RT-PCR and Western blot. Plasmid pEGFP-C1-PRL-3, pEGFP-C1-PRL-3(C104S) and pEGFP-C1-PRL-3(ΔCAAX) was generated by ligating BamH I/EcoR I digested full-length PRL-3, mutant PRL-3(C104S) and mutant PRL-3(ΔCAAX) to Bgl II/EcoR I digested pEGFP-C1 vector (Clontech, Palo Alto, CA).

### Immunofluorescence

To visualize green fluorescent protein (GFP) tagged PRL-3, BGC823 cells were transfected with pEGFP-C1, pEGFP-C1-PRL-3, pEGFP-C1-PRL-3(C104S) or pEGFP-C1-PRL-3(ΔCAAX). For immunofluorescence assays, BGC823 cells were transiently transfected and fixed with 4% paraformaldehyde for 10 min at room temperature, followed with DAPI(sigma) staining of 10 min. Cover slips were mounted on glass slides with 50% glycerol/phosphate-buffered saline and imaged using a Leica SP2 confocal system (Leica Microsystems, Dresden, Germany).

### Western blot

Cells were homogenized in lysis buffer (50 mM Tris–HCl, pH 7.5, 150 mM NaCl, 1% NP-40, 1 mM DTT, 1 mM phenylmethylsulfonyl fluoride, 10 mM NaF, 1 mM Na_3_VO_4_, 1 × protease cocktail) for 20 min at 4°C. The supernatant was collected after centrifugation at 12,000 × g for 20 min at 4°C and subjected to Western blot with GAPDH for the internal reference. PRL-3 antibody 3B6 was verified previously [25]. Documentation of blots was performed by scanning with an EPSON PERFECTION 2580 scanner and acquired images were adjusted by the Auto-Contrast command of Photoshop CS (Adobe, San Jose, CA).

### Motility and invasion assays

For transwell chamber-based motility and invasion assays, equal amounts of cells were loaded into an insert provided with serum-free medium and allowed to pass through an 8-μm-pore polycarbonate filter, which had been either pre-coated with 100 μg of Matrigel (Becton Dickinson, San Jose, CA) for invasion assay or left uncoated for motility assay. Medium supplemented with 10% fetal calf serum was added to the bottom chamber. Cells on the upper surface of filters were wiped out after 24 h (motility assay) or 48 h (invasion assay), and those on the undersurface were stained with 1% amino toluene blue and counted under a microscope.

### Statistical analysis

A standard chi-squared test was performed to assess the association between PRL-3 expression and the clinicopathological parameters. Survival curves were estimated by the Kaplan–Meier method and compared with the log rank test. Multivariate analysis was performed using the Cox regression model (a backward selection) to assess whether a factor was an independent predictor of disease free survival (DFS). Hazard ratios (HRs) with 95% confidence intervals (CIs) were estimated. A two tailed P-value of <0.05 was considered statistically significant. All statistical analyses were performed with SPSS v18.0 software (SPSS Inc., Chicago, IL, USA).

## Results

### Association of PRL-3 expression and clinicopathological factors

**Figure 1:**
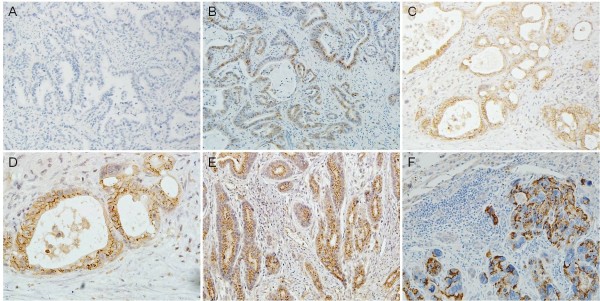
**Immunohistochemical staining of PRL-3 expression in gastric cancer. A**. Negative control; **B**. PRL-3 mild expression in gastric carcinoma (x200); **C**. PRL-3 moderate expression in gastric carcinoma (x200); **D**. Cellular location of PRL-3 expression, mainly localized in the cytomembrane with some granulated loci in cytoplasm (x400, magnification of **D**.); **E**. Intense expression of PRL-3 in the primary gastric carcinoma (x200); **F**. PRL-3 expression in matched liver metastasis developed 2 years after surgery (x200, **E** and **F** are from the same patient).

PRL-3 expression in 196 primary gastric tumor specimens and 21 cases of liver metastasis was determined by immunohistochemistry. As shown in Figure 1, PRL-3 protein mainly localized at cytomembrane and endomembrane systems, sometimes presented as granulated loci in the cytoplasm in the intensely positive samples (Figure 1). According to the criteria, positive expression(>10%) was found in 38 out of 196 neoplasms and 16 out of 21 liver metastasis (19.4% versus 76.2%, P < 0.001). In the 21 paired samples of primary cancer and liver metastasis, consistency of PRL-3 expression is observed with positive rate of 57.1% (12/21) and 76.2% (16/21), respectively (P < 0.05). Among them, we found one patient with positive PRL-3 expression developed liver metastasis 2 years after surgery, at that time no clinical detectable metastasis existed initially (Figure 1). Statistical analysis further showed positive associations of PRL-3 expression with lymph node involvement and vascular invasion (*P =* 0.000). Patients with lymph node status at N2 and N3 showed higher expression rates than those with lymph node status at N0 and N1 stage (27.8% (N2 + N3) versus 11.1% (N0 + N1), *P =* 0.006). Patients with positive vascular invasion also showed increased expression compared with those without (32.9% versus 11.4%, *P =* 0.000, Table 1). Likewise, we also observed a trend showing more elevated expression in the gastric cancer in advanced stages than in early stages [24.8% (stage III and IV) versus 12.0% (stage I and II)], or with distant metastasis than without distant metastasis (33.3% versus 17.7%), although there is no statistical significance (Table 1).

**Table 1:** Association of PRL-3 expression with clinicopathological parameters in gastric cancer

**Variables**	**Patients (n)**	**Expression of PRL-3**		**χ**^**2**^	***P***
		**Negative (%)**	**Positive (%)**		
**Age**					
<60	93	77 (82.8)	16 (17.2)	0.540	0.463
≥60	103	81 (78.6)	22 (21.4)		
**Sex**					
Male	146	123 (84.2)	23 (15.8)	4.837	0.128
Female	50	35 (70.0)	15 (30.0)		
Diagnosis*					
GC	177	145 (81.9)	32 (18.1)	2.001	0.217
GEC	19	13 (68.4)	6 (31.6)		
**TNM stage**					
I	33	29 (87.9)	4 (12.1)	6.170	0.100
II	50	44 (88.0)	6 (12.0)		
III	92	71 (77.2)	21 (22.8)		
IV	21	14 (66.7)	7 (33.3)		
**Tumor invasion**					
T1	20	17 (85.0)	3 (15.0)	1.654	0.659
T2	27	23 (85.2)	4 (14.8)		
T3	78	64 (82.1)	14 (17.9)		
T4	71	54 (76.1)	17 (23.9)		
**Lymph node metastasis**					
Negative	59	52 (88.1)	7 (11.9)	12.298	0.006
N1	40	36 (90.0)	4 (10.0)		
N2	34	28 (82.4)	6 (17.6)		
N3	63	42 (66.7)	21 (33.3)		
**Metastasis**					
Negative	175	144 (82.3)	31 (17.7)	2.927	0.138
Positive	21	14 (66.7)	7 (33.3)		
**Differentiation**					
Well	13	12 (92.3)	1 (7.7)	1.781	0.410
Moderately	89	69 (77.5)	20 (22.5)		
Poor	94	77 (81.9)	17 (18.1)		
**Histology**					
Adenocarcinoma	154	126 (81.8)	28 (18.2)	0.669	0.413
Others	42	32 (76.2)	10 (23.8)		
**Vascular invasion**					
Negative	123	109 (88.6)	14 (11.4)	13.543	0.000
Positive	73	49 (67.1)	24 (32.9)		

*GC: Gastric cancer; GEC: Gastroesophageal Cancer. Statistical analysis showed significant positive associations of PRL-3 expression with lymph node involvement and vascular invasion.

### PRL-3 expression predicted worse overcome in gastric cancer

**Figure 2:**
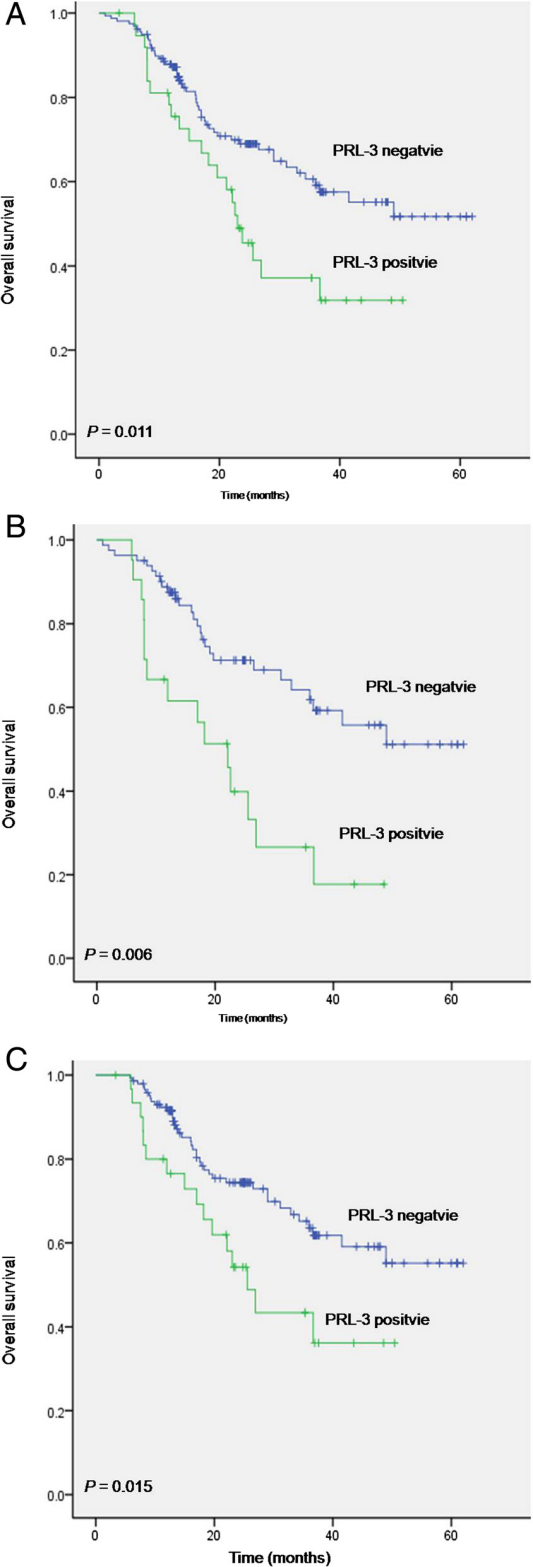
**Kaplan–Meier estimates of overall survival (OS) with respect to PRL-3 expression. A**. OS curves stratified by PRL-3 expression in gastric cancer tissues. **B**. OS curves stratified by PRL-3 expression in the subgroup of well and moderately differentiated carcinoma. **C**. OS curves for the subgroups of patients without distant metastasis stratified by PRL-3 expression.

As expected, clinical TNM stage, depth of tumor invasion, lymph node status, metastasis, vascular invasion and tumor location were significantly associated with clinical outcome (*P* < 0.01, Table 2). Patients with high level of PRL-3 expression exhibited significant poorer 5-year overall survival (OS) compared with patients with low level of PRL-3 (31.8% versus 51.7%, *P* = 0.011, Table 2, Figure 2A). A multivariate Cox proportional hazards model using variables associated with survival in our study (depth of wall invasion, lymph node metastasis, vascular invasion, distant metastasis, tumor location and PRL-3 expression) revealed that although the impact of PRL-3 on survival was less evident than vascular invasion (HR: 7.973, *P* = 0.011), tumor invasion (HR: 2.578, *P* = 0.020), and lymph node metastasis (HR: 5.520, *P* = 0.004), the risk of patients with positive PRL-3 expression dying from the disease was still 2.088 times higher than those with negative PRL-3 expression (*P* = 0.027). Thus, PRL-3 expression was an independent risk factor in gastric cancer outcome (Table 3).

**Table 2:** Univariate analysis of survival in gastric cancer according to clinicopathologic factors and PRL-3 expression

**Variables**	**Patients (n)**	**5-year survival**	***P***
		**(% ± SD)**	
**Age**			
<60	93	65.3% ± 0.056	0.716
≥60	103	63.1% ± 0.052	
**Sex**			
Male	146	81.5% ± 0.034	0.764
Female	50	50.3% ± 0.082	
Diagnosis*			
GC	177	83.1% ± 0.029	0.006
GEC	19	10.6% ± 0.092	
**TNM stage**			
I	33	74.3% ± 0.078	0.000
II	50	53.1% ± 0.239	
III	92	52.1% ± 0.060	
IV	21	15.6% ± 0.082	
**Tumor invasion**			
T1	20	50.0% ± 0.354	0.000
T2	27	53.1% ± 0.225	
T3	78	53.8% ± 0.068	
T4	71	35.3% ± 0.071	
**Lymph node metastasis**			
Negative	59	55.4% ± 0.145	0.000
N1	40	69.1% ± 0.091	
N2	34	18.2% ± 0.064	
N3	63	24.2% ± 0.075	
**Metastasis**			
Negative	175	86.1% ± 0.027	0.000
Positive	21	15.6% ± 0.082	
**Differentiation**			
Well	13	75.2% ± 0.126	0.278
Moderately	89	38.1% ± 0.075	
Poor	94	55.2% ± 0.065	
**Histology**			
Adenocarcinoma	154	43.7% ± 0.057	0.191
Others	42	65.0% ± 0.091	
**Vascular invasion**			
Negative	123	58.3% ± 0.068	0.000
Positive	73	37.0% ± 0.063	
**PRL-3**			
Negative	114	51.7% ± 0.061	0.011
Positive	82	31.8% ± 0.090	

*GC: Gastric cancer; GEC: Gastroesophageal Cancer. As expected, clinical TNM stage, depth of tumor invasion, lymph node status, metastasis, vascular invasion and tumor location were significantly associated with clinical outcome.

**Table 3:** Multivariate analysis of survival in gastric cancer according to clinicopathologic factors and PRL-3 overexpression

	**Overall survival**			
**Variables**	**HR**	**95% CI**		***P***
		**Lower**	**Upper**	
**PRL-3**	2.088	2.010	2.759	.027
**Tumor invasion**	2.578	1.159	5.732	.020
**Lymph node metastasis**	5.520	1.714	17.774	.004
**Tumor size**	1.023	1.001	1.455	.083
**Vascular invasion**	7.973	1.608	39.539	.011

Multivariate analysis showed PRL-3 expression was an independent risk factor in gastric cancer outcome.

To further analyze the prognosis potential of PRL-3 in gastric cancer, patients were divided into subgroups according to differentiation. In the subgroup of well and moderately differentiated patients, PRL-3 expression was significantly associated with overall survival (59.2% versus 17.7%, P = 0.001, Figure 2B). Also, in the subgroup of unmetastatic gastric cancer, patients with PRL-3 expression showed worse outcome compared with those did not express PRL-3(36.1% versus 55.2%, *P* = 0.015, Figure 2C), while there is no significant difference in the metastatic subpopulation.

### Construction of wild type PRL-3 and mutant PRL-3 protein expression vectors and establishment of stable cell pools with BGC823

To investigate the biological functions of PRL-3, we constructed wild-type (WT) and mutant PRL-3 fusion expression vectors. The mutant Myc-PRL-3(C104S) vector was consisted of an inactivating mutation of the essential catalytic cysteine to serine at position 104 in PRL-3 tyrosine phosphatase (PTP) signature motif (VHCX-AGXXR), which could abolish its PTP activity. The mutant Myc-PRL-3(ΔCAAX) are constructed without the CAAX prenylation motif in the C-terminal, recognization of which help the correct localization to specific sites within the cells and further enables participation in their relevant signal pathway.

**Figure 3:**
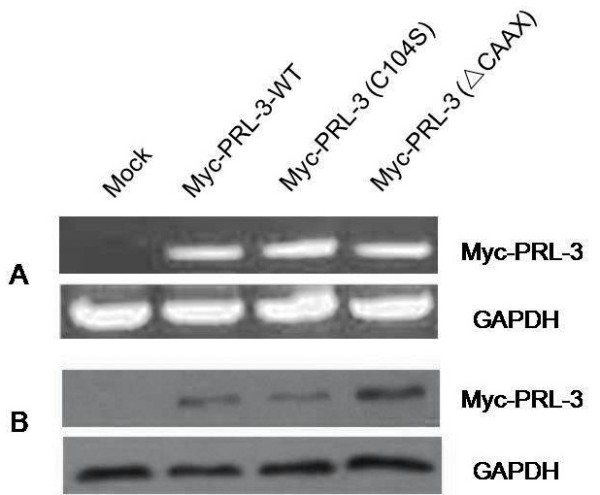
**Level of Myc-PRL-3-WT, Myc-PRL-3(C104S), Myc-PRL-3(**Δ**CAAX) expression in stable BGC823 cell pools.** They are detected by RT-PCR **(A)** and Western blot **(B)**. Results shown are representative of three independent experiments.

**Figure 4:**
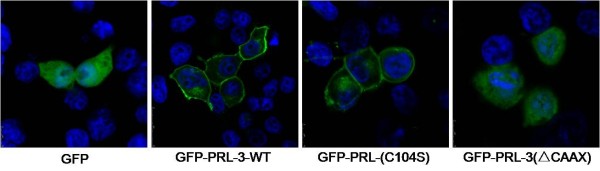
**Immunofluorescence of wild type PRL-3 and its mutants.** Intracellular localization of PRL-3. BGC823 cells were transfected with pEGFP-C1-PRL-3-WT, pEGFP-C1-PRL-3(C104S) and pEGFP-C1-PRL-3(ΔCAAX) expression vectors. Nuclei were stained with 4, 6-diamidino-2-phemylindole (blue). PRL-3 expression was presented as green fluorescence.

The stable BGC823 cell pools expressing Myc-PRL-3-WT, mutant Myc-PRL-3(C104S) and Myc-PRL-3(ΔCAAX) were then obtained with transfection and Geneticin selection. RT-PCR and WB verified their expression (Figure 3). Together, The wild type EGFP-PRL-3, its mutant EGFP-PRL-3(C104S) and EGFP-PRL-3(ΔCAAX) vectors were created as described and transiently transfected into BGC823 cells. The subcellular localization of PRL-3 and its mutants were observed by immunofluorescene (Figure 4). The wild type EGFP-PRL-3 existed in the plasma membranes and some intracellular structures in the cytoplasm. The catalytic inactive mutation in EGFP-PRL-3(C104S) did not appear to change the subcellular localization and membrane association. In contrast, the mutant EGFP-PRL-3(ΔCAAX) was mostly found within the cytoplasm and nuclear.

### Metastatic ability of BGC823 cells expressing wild type Myc-PRL-3 or mutants

**Figure 5:**
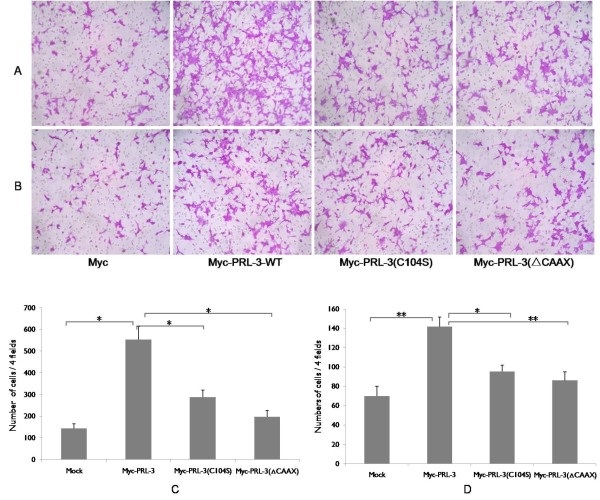
**Effects of PRL-3-WT/mutations on migration and invasion. A**. Migration assay in BGC823 cells expressing Myc-PRL-3-WT, mutant Myc-PRL-3(C104S) and Myc-PRL-3(ΔCAAX) using transwell chamber; **B**. Invasion assay in BGC823 cells expressing Myc-PRL-3-WT, mutant Myc-PRL-3(C104S) and Myc-PRL-3(ΔCAAX). Quantitative analysis of the number of the cells migrated to the lower side of the membrane in migration assay **(C)** and invasion assay **(D)** is shown. All Data are mean ± SE of three independent experiments. *, P < 0.05; **, P < 0.01.

The prometastatic capabilities of PRL-3 were analyzed by transwell chamber in BGC823 cells stably expressing Myc-PRL-3 fusion proteins or its mutants. Myc-PRL-3-WT expressing BGC823 cells resulted in a 3.8- and 2.0-fold, respectively, enhanced migration and invasion to the under surface compared to control that transfected with mock (Figure 5). However, Cells expressing Myc-PRL-3(C104S) had such effects reduced significantly by 48% and 32% compared with wild type PRL-3 on cellular migration or invasion, respectively (Figure 5). Likewise, we observed the increased motility and invasion abilities were also greatly crippled by 64% and 39% once CAAX motif deleted, suggesting the important impact of subcellular location for the biological function of PRL-3.

## Discussion

PTPs play a fundamental role in regulating protein phosphorylation balance and PRL-3 represent as a member of a new class of PRL superfamily [2]. In recent years, PRL-3 expression has been evaluated in various human cancers and found to be associated with invasion, metastasis, and poor prognosis [5,15,26]. In this report, we found significant positive association of PRL-3 expression with lymph node metastasis and vascular invasion. Patients with distant metastasis or in the advanced stage also exhibited higher PRL-3 expression, suggesting it as a biomarker for tumor metastasis and aggressiveness. In previous studies, Miskad et al. were the first to describe the role of PRL-3 protein in gastric cancer [11]. Using polyclonal antibody, they showed that PRL-3 is positively correlated with lymph node metastasis and tumor stage. In the study carried out by Wang et al., PRL-3 expression was more frequently detected in the lymph node metastasis than in the matched primary tumor [13]. Our results are consistent with these literatures, but with a relatively lower positive rate (19.4%) considering that monoclonal antibody was utilized in this study to exclude the possibility for crossacting with the other PRL families. Furthermore, PRL-3 expression in 21 cases of liver metastasis was reported here for the first time, the positive rate is 76.2% compared with 57.1% in the matched primary lesions. Thus, high level of PRL-3 overexpression is observed in primary gastric tumors, higher in primary tumors with metastases, and the highest in liver metastatic tumors. This indicates the specific involvement of PRL-3 protein in the metastatic process. As expected, survival analysis showed that patients with PRL-3 positive expression has a significant worse overall survival compared with those do not express. Importantly, in the subgroup analysis, we observed that PRL-3 expression significantly distinguished patients’ survival in the population without metastasis, also in the patients with well to moderately differentiated gastric cancer. This facts may suggest that PRL-3 could serve as a prognostic factor for predicting poorer outcome, but not in the late stage when numerous deregulations have accumulated to the extent that a single molecule is not enough to explain the overall state of disorder [27]. In light of the evidence discussed here, we propose that PRL-3 is a key metastasis-initiating gene deregulated early in the metastatic process, driving metastasis progression from primary to distant sites via lymphatic or blood circulation. The different positive rates were reported probably due to the distinguished clones of antibody used, distinct evaluation criteria or the different population of patients involved.

As a phosphatase, PRL-3 has a conserved core PTP domain with the signature C(X)5R active site motif. Meanwhile, PRL family is known to bear the membrane-targeting CAAX prenylation motif at COOH-terminus [28]. In order to explore the relationship between the catalytic activity and subcellular localization of PRL-3 with its prometastatic function, we constructed PRL-3 wild type, its catalytic inactive mutant (C104S) and CAAX motif deleted mutant (ΔCAAX) vector and investigated their effects on cell migration and invasion *in vitro*. Compared to the control group, BGC823 cells transfected with PRL-3-WT exhibited significant elevated ability of migration and invasion. However, both the mutants have greatly abolished this phenomenon, especially for the mutant PRL-3(ΔCAAX) had its ability decreased to a more significant degree. The defeat of PRL-3(C104S) mutant could be explained by its loss of phosphatase activity or a potential to form an inter- molecular disulfide bond to act on its downstream targets, which is also observed in colon cancer in Guo’s study [29]. It is therefore hypothesized that both the phosphatase catalytic activity and its cytomembrane location is indispensable for its function in gastric cancer metastasis. The process may involve interaction in the signalling pathway on the inner side of the membrane. Actually, by using a yeast two-hybrid system, our previous study has identified integrin α1 on cell membrane as a PRL-3-interacting protein [17], and reduced the phosphorylation level of integrin β1, hence activating the MAPK pathway and promoting colon cancer metastasis *in vitro* and *in vivo*[4].

Although many proteins with the CAAX family depend on such modifications for correct location, they can be targeted to different subcellular sites [30]. In our present study by immunofluorescent microscopy, GFP-PRL-3-WT and GFP-PRL-3(C104S) fusion proteins were localized to cytomembrane and some intracellular structures in the cytoplasm, while the GFP-PRL-3(ΔCAAX) mutation resulted in the diversion of the majority of the protein to the cytoplasma and nuclear. In the detection of gastric cancer tissue samples with immunohistochemistry, we also observed its location mainly at cytomembrane and endomembrane system. Previous study has reported that PRL-3 are normally associated with the cytoplasmic face of the plasma membrane and other plasma membrane processes such as endosome [31]. Though the exact subcellular localization of PRL-3 was not investigated in this work, our current data suggested, at least that PRL-3 could locate on the plasma membrane in gastric cancer cells and further, CAAX motif was the key component for its localization while cysteine at 104 was not influence its distribution. These results are consistent with some previous studies, which found that overexpression of HA-PRL-3 in colon cancer cells was presented as cell plasmic membrane localization [5,32], or in the membrane ruffles, protrusions and some vacuolar-like membrane extensions [24,33]. But nuclear localization of PRL-3 has also been reported. These controversial results may be partially explained by the hypothesis that PRL-3 could shuttle between the nucleus and cytoplasm. The reasons partly come from PRL-1, another member of the PRL superfamily. PRL-1 was reported acting in a prenylation-dependent manner in the interphase while regulating its spindle dynamics in a prenylation-independent manner in the mitotic phase, and finally take functions in cell survival and motility [34,35]. In present study, we found that deletion of the C-terminus prenylation motif of PRL-3 promotes their cytoplasma and nuclear accumulation. There is possibility that reversible prenylation could regulate PRL-3 nucleo-cytoplasmic distribution and exert different functions, which further researches are still needed. In fact, many proteins containing the CAAX family are also oncogenes, such as Ras and Rho superfamily [36,37]. For this reason, investigations into the mechanisms of farnesylation and prenylation transferase inhibitors are becoming a potential new generation of agents for anticancer treatment [38,39].

## Conclusions

In summary, despite substantial advances in cancer therapy, metastatic disease remains the primary cause of death in gastric cancer. PRL-3 is one of the numerous genes that have been directly linked to the process. Our study here indicated that the metastasis associated protein PRL-3 could be a independent prognostic factor for predicting worse outcome in gastric cancer. Both its catalytic activity and CAAX motif for its intracellular localization are critical for its prometastatic capability, which shedding new light for further investigation on its downstream pathway. PRL-3 is becoming increasingly attractive for personalized cancer therapy for metastatic intervention.

## Abbreviations

PRL-3: Phosphatase of regenerating liver-3; PTP: Protein tyrosine phophatases; SAGE: Serial analysis of gene expression; IHC: Immunohistochemistry staining; AJCC: American joint committee on cancer; GAPDH: Glyceraldehyde-3-phosphate dehydrogenase; PBST: Phosphate buffered saline add Tween-20; OS: Overall survival; HR: Hazard ratio; CI: Confidence interval.

## Competing interests

The authors declare that they have no competing interests.

## Authors’ contributions

XXF and LSY conceived the study, performed most experiment and drafted the manuscript. HY participated in collecting the specimens. LZY and ZLH participated in the clinical data collection. WXZ participated in the manuscript writing and revision. DH carried out celluar studies. ML construceted the expression vector. JYN carried out a part of celluar studies. SCC and JJF conceived the study, participated in its design and gave final approval of the version to be published. All authors read and approved the final manuscript.
