# Correction: Phosphatase of regenerating liver-3 (PRL-3) is associated with metastasis and poor prognosis in gastric carcinoma

**DOI:** 10.1186/1479-5876-12-73

**Published:** 2014-03-26

**Authors:** Xiaofang Xing, Shenyi Lian, Ying Hu, Ziyu Li, Lianhai Zhang, Xianzi Wen, Hong Du, Yongning Jia, Zhixue Zheng, Lin Meng, Chengchao Shou, Jiafu Ji

**Affiliations:** 1 Department of gastrointestinal translational research, Peking University, Peking, China; 2 Department of Biochemistry and Molecular Biology, Peking, China; 3 Tissue bank, Key laboratory of Carcinogenesis and Translational Research (Ministry of Education) Peking University Cancer Hospital & Institute, Beijing, China; 4 Department of gastrointestinal surgery, Key laboratory of Carcinogenesis and Translational Research (Ministry of Education) Peking University Cancer Hospital & Institute, Beijing, China

## Text

After publication of this work [1], we found that we inadvertently failed to include the complete title of the affiliation. The full description of affiliation has now been added and the Authors’ contributions and Competing interests section modified accordingly.

## Competing interests

The authors declare that they have no competing interests.

## Authors’ contributions

XXF and LSY conceived the study, performed most experiment and drafted the manuscript. HY participated in collecting the specimens. LZY and ZLH participated in the clinical data collection. WXZ participated in the manuscript writing and revision. DH carried out celluar studies. ML construceted the expression vector. JYN carried out a part of celluar studies. SCC and JJF conceived the study, participated in its design and gave final approval of the version to be published. All authors read and approved the final manuscript.
